# Review: systematic review of effectiveness of art psychotherapy in children with mental health disorders

**DOI:** 10.1007/s11845-021-02688-y

**Published:** 2021-07-06

**Authors:** Irene Braito, Tara Rudd, Dicle Buyuktaskin, Mohammad Ahmed, Caoimhe Glancy, Aisling Mulligan

**Affiliations:** 1 School of Medicine, University College Dublin, Dublin, Ireland; 2 Paediatric Medicine, Great North Children’s Hospital, United Kingdom, UK; 3 Dublin North City and County Child and Adolescent Mental Health Service, Dublin, Ireland; 4 Department of Child and Adolescent Psychiatry, Cizre Dr. Selahattin Cizrelioglu State Hospital, Cizre, Sirnak, Turkey; 5 Department of Child and Adolescent Psychiatry, University College Dublin, Dublin, Ireland

**Keywords:** Adolescent, Art therapy, CAMHS, Child, Mental health, Psychotherapy

## Abstract

Art therapy and art psychotherapy are often offered in Child and Adolescent Mental Health services (CAMHS). We aimed to review the evidence regarding art therapy and art psychotherapy in children attending mental health services. We searched PubMed, Web of Science, and EBSCO (CINHAL®Complete) following PRISMA guidelines, using the search terms (“creative therapy” OR “art therapy”) AND (child* OR adolescent OR teen*). We excluded review articles, articles which included adults, articles which were not written in English and articles without outcome measures. We identified 17 articles which are included in our review synthesis. We described these in two groups—ten articles regarding the treatment of children with a psychiatric diagnosis and seven regarding the treatment of children with psychiatric symptoms, but no formal diagnosis. The studies varied in terms of the type of art therapy/psychotherapy delivered, underlying conditions and outcome measures. Many were case studies/case series or small quasi-experimental studies; there were few randomised controlled trials and no replication studies. However, there was some evidence that art therapy or art psychotherapy may benefit children who have experienced trauma or who have post-traumatic stress disorder (PTSD) symptoms. There is extensive literature regarding art therapy/psychotherapy in children but limited empirical papers regarding its use in children attending mental health services. There is some evidence that art therapy or art psychotherapy may benefit children who have experienced trauma. Further research is required, and it may be beneficial if studies could be replicated in different locations.

## Introduction

Child and Adolescent Mental Health Services (CAMHS) often offer art therapy, as well as many other therapeutic approaches; we wished to review the literature regarding art therapy in CAMHS. Previous systematic reviews of art therapy were not specifically focused on the effectiveness in children [1–5] or were focused on the use of art therapy in children with physical conditions rather than with mental health conditions [6]. The use of art or doodling as a communication tool in CAMHS is long established—Donald Winnicott famously used “the Squiggle Game” to break boundaries between a patient and professional to narrate a story through a simple squiggle [7]. Art is particularly useful to build a rapport with a child who presents with an issue that is too difficult to verbalise or if the child does not have words to express a difficulty. The term art therapy was coined by the artist Adrian Hill in 1942 following admission to a sanatorium for the treatment of tuberculosis, where artwork eased his suffering. “Art psychotherapy” expands on this concept by incorporating psychoanalytic processes, seeking to access the unconscious. Jung influenced the development of art psychotherapy as a means to access the unconscious and stated that “by painting himself he gives shape to himself” [8]. Art psychotherapy often focuses on externalising the problem, reflecting on it and analysing it which may then give way to seeing a resolution.

The UK Joint Commissioning Panel for Mental Health 2013 recommends that psychotherapists and creative therapists are part of the CAMHS teams [9]. There is a specific UK recommendation that art therapy may be used in the treatment of children and young people recovering from psychosis, particularly those with negative symptoms [10], but no similar recommendation in the Irish HSE National Clinical Programme for Early Intervention in Psychosis [11]. There is less clarity about the use of art therapy in the treatment of depression in young people—arts therapies were previously recommended [12], but more recent NICE guidelines appear to have dropped this advice, though the recommendation for psychodynamic psychotherapy has remained [13]. Art therapy is often offered to treat traumatised children, but we note that current NICE guidelines on the management of PTSD do not include a recommendation for art therapy [14]. The Irish document “Vision for Change” did not include a recommendation regarding art psychotherapy or creative therapies [15]. Similarly, the document “Sharing the Vision” does not make any recommendation regarding creative or art therapies, though it recommends psychotherapy for adults and recommends arts activities as part of social prescribing for adults [16]. Meanwhile, it is not uncommon for there to be an art therapist in CAMHS inpatient units, working with those with the highest mental healthcare needs. We wished to find out more about the evidence for, or indeed against, the use of art therapy in CAMHS. We performed a systematic review which aimed to clarify if art psychotherapy is effective for use in children with mental health disorders. This review aimed to address the following questions: (1) Is art therapy/psychotherapy an effective treatment for children with mental health disorders? (2) What are the various methods of art therapy or art psychotherapy which have been used to treat children with mental health disorders and how do they differ in terms of (i) setting and duration, (ii) procedure of the sessions, and (iii) art activities details?

## Methods

### Procedure

The Preferred Reporting Items for Systematic Reviews (PRISMA) statement for systematic reviews was followed. Searches and analysis were conducted between September 2016 and April 2020 using the following databases: PubMed, Web of Science and EBSCO (CINHAL®Complete). The following “medical subject terms” were utilized for searches: (“creative therapy” OR “art therapy”) AND (child* OR adolescent OR teen*). Review publications were excluded. Studies in the English language meeting the following inclusion criteria were selected: (i) use of art therapy/art psychotherapy, (ii) psychiatric disorder/diagnosis and/or mood disturbances and/or psychological symptoms, (iii) human participants aged 0–17 years inclusive. Articles investigating the efficiency of art therapy in children with medical conditions were included only if the measured outcome related to psychological well-being/symptoms. Exclusion criteria included: (i) application of therapies which do not involve art activities, (ii) application of a combination of therapies without individual results for art therapy, (iii) not clinical studies (review, meta-analysis, reports, others), (iv) studies which focused on the artwork itself/art therapy procedure and did not measure and publish any clinical outcomes, (v) absence of any pre psychiatric symptoms or comorbidity in the participant sample prior to art intervention. All articles were screened for inclusion by the authors (MA, TR, IB, AM, DB), unblinded to manuscript authorship.

### Data extraction

The authors (IB, TR, AM, MA, DB) extracted all data independently (unblinded). Data were extracted and recorded in three tables with specific information from each study on (i) the study details, (ii) art therapy details and outcome measures and (iii) art therapy results. The following specific *study details* were extracted: author/journal, country, year of publication, study type (i.e. study design), study aims, study setting, participant details (number, age and gender), disease/disorder studied and inclusion criteria and exclusion criteria of the study. The following details were extracted regarding the *art therapy provided and outcome measures*: type of art therapy provided (individual or group therapy), the art therapy procedure and/or techniques used, the art therapy setting, therapy duration (including frequency and duration of each art therapy session), the type of outcome measure used, the investigated domains, the time points (for outcome measures) and the presence or absence of pre-/post-test statistical analysis. Finally, we extracted specific information on the *art therapy results*, including therapy group results, control group results, the number and percentage of who completed therapy, whether or not a pre-/post-test statistical difference was found and the general outcome of each study. Following the extraction of all data, studies included were divided into two groups: (1) children with psychiatric disorder diagnosis and (2) children with psychiatric symptoms. Finally, the QUADAS-2 tool was used to assess the risk of bias for each study, and a summary of the risk of bias for all data was calculated [17]. The QUADAS-2 is designed to assess and record selection bias, performance bias, detection bias, attrition bias, reporting bias and any other bias [17].

## Results

### Study inclusion and assessment

**Fig. 1:**
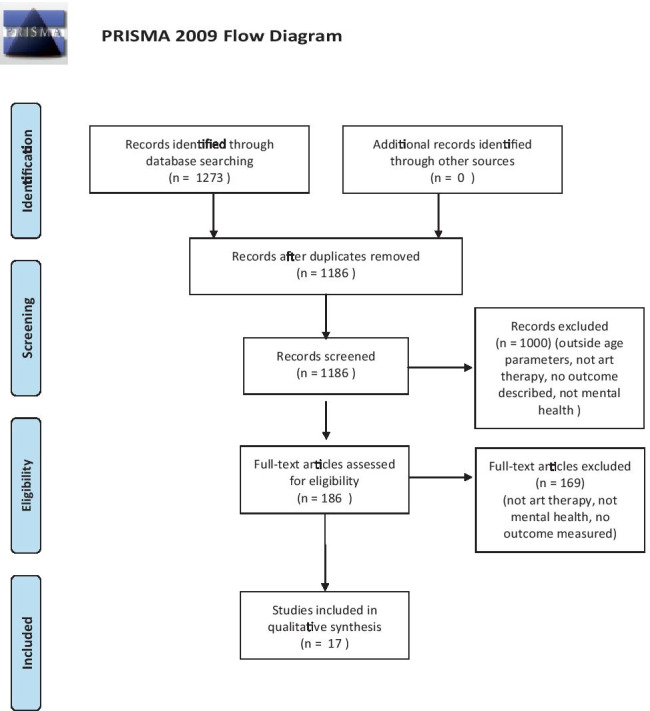
PRISMA 2009 flow diagram

A total of 1273 articles were initially identified (Fig. 1). After repeats and duplicates were removed, 1186 possible articles were identified and screened for inclusion/exclusion according to the title and abstract, which resulted in 1000 articles being excluded. The remaining 186 full articles were retrieved and full text considered. Following review of the full text, 70 articles were selected and further analysed. Fifty-three of them did not meet our criteria for review. Reasons for exclusion were grouped into four main categories: (1) not art therapy [*n* = 2]; (2) not mental health [*n* = 5]; (3) no outcome measured [*n* = 18]; (4) other reasons (i.e. descriptive texts, full article not available) [*n* = 28]. In conclusion, there were 17 articles remaining that met the full inclusion criteria, and further descriptive analysis was performed on these 17 studies. All the considered articles were produced in the twenty-first century, between 2001 and 2020, most in the USA (60%), followed by Canada (30%) and Italy (10%). The characteristics of studies included in our final synthesis are reported in Tables 1 and 2.

**Table 1:** Children with psychiatric disorder diagnosis

Art therapy details										
			Art therapy description			Outcome measures				
Author	Type	Procedure	Duration	Setting	Type	Investigated domains	Time Points	Pre-/post-test statistical analysis	*N*	Study design
Chapman et al. [18]	One-to-one session	1. Graphic kinaesthetic activity; 2. Series of drawings; 3. Verbal narrative	1 h, frequency not reported	Hospital room, bedside	PTSD-I Child or Adolescent Version	Self-report 20-item inventory of symptoms based primarily on the DSM-IV diagnostic criteria	1 week, 1 month	Mean % change	85	Randomised controlled trial
Mallay [29]	Individual	Were incorporated	Not reported	Children’s home	Therapist’s analysis of the sessions	Concepts of emotions, thoughts, family system dynamics post-event	After each session, and at the end of the therapy	NA	1	Case Study
Henley [23]	Individual art therapy	Type 1: pre-art making discussion + art session; Type 2: art session + post-art making reflection	Long-term therapy with as-needed basis sessions	NA	Therapist’s analysis of the sessions	NA	After each session, and at the end of the therapy	NA	4	Case Series
Briks [22]	Individual art therapy	NA	2 weekly sessions for 1 year, then 1 weekly session for 2 years 11 months	Out-patient psychiatric unit	Therapist’s analysis of the sessions	NA	After each session, and at the end of the therapy	NA	1	Case Study
Lyshak-Stelzer et al. [19]	Trauma-focused group art therapy (*n* = 2–5)	(1) 5–20-min discussion about the topic of the day, (2) art-making period, (3) verbal narrative	1 h weekly group session over 16 weeks	In-patient	University of California at Los Angeles (UCLA) PTSD Reaction Index	22 self-report items based on: (1) DSM-IV PTSD symptom criteria; (2) avoidance symptoms; (3) hyperarousal symptoms	Before and after treatment	ANOVA analysis	29	Randomised trial Compared art therapy vs TAU art making
McCullough [24]	Individual art therapy	Tailored art therapy program with open studio sessions, art directives, family work, and/or verbal therapy techniques	45-min weekly session over 8 months	Out-patient facility	Therapist’s analysis of the sessions	NA	After each session, and at the end of the therapy	NA	1	Case Study
Shore [27]	Individual art therapy	NA	NA	NA	Therapist’s analysis of the sessions	NA	After each session, and at the end of the therapy	NA	1	Case Study
Gatta et al. [21]	Group Art Therapy	(1) Welcoming + 15-min conversation about the previous week; (2) 5-min music; (3) 40-min art workshop while listening the music; (5) 20-min verbal narrative; (6) 10-min conclusion of the session	1st cycle: 8 sessions (May-Jun 2012) 2nd cycle: 10 sessions (Sep–Dec 2012). Each patient is requested to attend at least once during each cycle	In-patient	MacKenzie’s Group Climate Questionnaire	12 items specialist-report based on (1) involvement; 2() conflict; (3) avoidance	After the first and the second cycle	*t* test unpaired	9	Quantitative and qualitative quasi-experimental
Lee [25, 26]	Individual art therapy	(1) AT session; (2) video-recording watching session; (3) semi-structured interview with video-stimulated recall	NA	Outpatient, family therapy service	Therapist’s analysis of the sessions (videotapes, notes, post-session interviews)	(1) Acculturation gap themes (role reversal, communication challenges, locked out of opportunities); flow indicator themes (access to diverse media, self-assignment, self-correction)	After each session, and at the end of the therapy Cross-case analysis was provided	NA	3 3	Qualitative multi-case study Qualitative multi-case study

*PTSD-I* post-traumatic stress disorder index, *DSM-IV* diagnostic and statistical manual of mental disorders IV, *PTSD* post-traumatic stress disorder, *CI* confidence interval, *ANOVA* analysis of variance, *AT* art therapy

**Table 2:** Children with psychiatric symptoms

Art therapy details										
			Art therapy description			Outcome measures				
Author	Type	Procedure	Duration	Setting	Type	Investigated domains	Time points	Pre-/post-test statistical analysis	*N*	Study design
Favara-Scacco et al. [28]	Individual art therapy	(1) Clinical dialogue; (2) visual imagination; (3) medical play; (4) structured drawing; (5) free drawing; (6) dramatization	Single session before the painful procedure	In-patient	Patient’s behaviour observation	15 positive behaviours as indicators of 4 main domains: cooperation; active compliance; passive compliance; good anxiety tolerance	Before, during and after a painful procedure	NA	32	IIa Non-randomised controlled
Kearns [30]	Individual art therapy	(1) Brief verbal check-in; (2) art or pre-art activity (oil-based clay work, finger-painting, and easel painting)	20-min sessions over 10 weeks. Art therapy sessions are alternate with days without art therapy	Kindergarten	(1) PPAT; (2) teacher’s behavioural report	(1) PPAT: colour, design, details and realism of the artwork; implied energy; developmental level; (2) behavioural report: positive and negative behaviours (recorded every 30 min)	Pre- and post-intervention	NA	1	III Case Study within case
Higenbottam [31]	Group art therapy	(1) Thematic beginning (“sign-in”); (2) group discussion about the events experienced in the week before; (3) group art activity (spontaneous or directives either)	1 h and a half weekly session, for 8 weeks	School	Daley & Lecroy’s Go Grrrls Questionnaire (modified with permission)	Body-image, self-concept, self-confidence, current cultural role models and influences	Pre- and post-intervention	Wilcoxon signed-ranks test	7	III Pre and post measures
Hartz and Thick [32]	Group art therapy	Magazine collage and yarn basket-making Art psychotherapy: psychoeducational presentation + encouraged abstraction, symbolization, and verbalization Art as therapy: focused on design potentials, technique, and the creative problem-solving process	Ten 1-h and a half art therapy sessions over a 12-week period	Juvenile facility	(1) SPPA; (2) Hartz AT-SEQ	SPPA: 45 items self-esteem measure (Scholastic/Job Competence, Social Acceptance, Athletic Competence, Physical Appearance, Behavioural Conduct, Global Self-Worth); Hartz AT-SEQ: 20 questions questionnaire (development of mastery, social connection, and self-approval)	SPPA: pre- and post intervention; Hartz AT-SEQ: post-intervention	Fisher’s t	27	IIb Quasi-experimental design
Darewych [33]	Individual art therapy: single-subject/AB experimental design	(1) Established baseline (A) = verbal sessions; (2) Implemented treatment (B) = art psychotherapy (name poster/sculpture, a house-tree-person, three wishes, a self-mask body tracing, a “me”-box, collage)	Group A = two 1-h verbal sessions + six 1-h art therapy sessions; (1) Group B = four 1-h verbal sessions + six 1-h art therapy sessions	In-patient and dwelling environment	(1) CDI; (2) TSCS:2; (3) Direct Measurement Rating Sheet	CDI: 27-item scale self-report symptom-orientated (Mood, Interpersonal Problems, Effectiveness level, Self Esteem); TSCS:2: 20-items measure about self-concept; Direct Rating: communication, self-esteem, self-concept, depression, development, and problem solving	CDI and TSCS:2: pre- and post-intervention; Direct Rating: after each session	NA	6	IIb Quasi-experimental design
Coholic and Eys [34]	Group art therapy	(1) 10-min opening activity; (2) art session (arts-based mindfulness methods) + 15 min break mid-way; (3) closing activity	Twelve weekly 2-h sessions	NA	Quantitative analysis: (1) Piers-Harris 2; (2) RSCA; Qualitative analysis: (1) semi-structured individual interviews	(1) Piers-Harris 2: 60 test items (overall self-concept; behavioural adjustment; intellectual and school status; physical appearance and attributes; freedom from anxiety; popularity; happiness and satisfaction); (2) RSCA: 64 items test (sense of mastery; sense of relatedness; emotional reactivity)	Pre- and post-intervention	MANOVA	77	IIb Repeated-measures observation
Siegel et al. [29]	Individual art therapy	Co-creating healing sock creatures art therapy	Single 90-min session	Hospital	Self-Report Mood Questionnaire	Emotional responses	Before and after the therapy period	2-tailed Student’s *t* test analysis	25	IIa WL controlled

*PPAT* person picking an apple from a tree, *SPPA* Self-Perception Profile for Adolescents, *Hartz AT-SEQ* Hartz Art Therapy Self-Esteem Questionnaire, *CDI* Children’s Depression Inventory, *TSCS:2* Tennessee Self Concept Scale: Short Form, *Piers-Harris 2* Piers-Harris Children’s Self-Concept Scale, *RSCA* Resiliency Scales for Children and Adolescents, *MANOVA* multivariate analysis of variance

### Participant characteristics

Participants in the 17 studies ranged from 2 to 17 years old inclusive. In ten articles, children with an *established psychiatric diagnosis* were included (Group 1, see Table 1). The type of psychiatric disorders as (i) PTSD, (ii) mood disorders (bipolar affective disorder, depressive disorders, anxiety disorder), (iii) self-harm behaviour, (iv) attachment disorder, (v) personality disorder and (vi) adjustment disorder. In seven articles, children with *psychiatric symptoms* were enrolled, usually referred by practitioners and school counsellors (Group 2, see Table 2). Participants had a wide variety of conditions including (i) symptoms of depression, anxiety, low mood, dysthymic features; (ii) attention and concentration disorder symptoms; (iii) socialisation problems and (iv) self-concept and self-image difficulties. Some children had medical conditions such as leukaemia requiring painful procedures, or glaucoma, cancer, seizures, acute surgery; others had experienced adversity such as parental divorce, physical, emotional and/or sexual abuse or had developed dangerous and promiscuous social habits (drugs, prostitution and gang involvement).

### Study design: children with an established psychiatric diagnosis (Table 1)

A summary of the ten studies on art therapy in children with a psychiatric diagnosis can be seen in Table 1, with further information about each study. There are just two randomised controlled in this category, both treating PTSD in children [18, 19]. Chapman et al. [18] provided individual art therapy to young children who had experienced trauma and assessed symptom response using the PTSD-I assessment of symptoms 1 week after injury and 1 month after hospital admission [18]. Their study included 85 children; 31 children received individual art therapy, 27 children received treatment as usual and 27 children did not meet criteria for PTSD on the initial PTSD-I assessment [18]. The art therapy group had a reduction in acute stress symptoms, but there was no significant difference in PTSD scores [18]. The second randomised controlled trial provided trauma-focused group art therapy in an inpatient setting and showed a significant reduction in PTSD symptoms in adolescents who attended art therapy in comparison to a control group who attended arts-and-crafts. However, this study had a high drop-out rate, with 142 patients referred to the study and just 29 patients who completed the study [19].

The remaining studies regarding art therapy or art psychotherapy in children with psychiatric disorders are case studies, case series or quasi experimental studies, most with less than five participants. All these studies reported positive effects of art therapy; we did not find any published negative studies. We can summarise that the studies differed greatly in the type of therapy delivered, in the setting (group or individual therapy) and in the types of disorders treated (Table 1).

### Forms of art therapy intervention and assessment (Table 1)

The various modalities and duration of art therapy described in the ten studies with children with psychiatric diagnoses are summarised in Table 1. The treatment of PTSD was described in two studies, but each described a different art therapy protocol, and the studies varied in terms of setting and duration [18, 19]. The Trauma Focused Art Therapy (TF-ART) study described 16 weekly in-patient group sessions [19], whereas the Chapman Art Therapy Treatment Intervention (CATTI) is a short-term individual therapy, lasting 1 h at the bedside of hospital inpatients [18]. Despite the differences, the methods have some common aspects. Both therapy methods focused on helping the individual express a narrative of his/her life story, supporting the individual to reflect on trauma-related experiences and to describe coping responses. Relaxation techniques were used, such as kinaesthetic activities [18] and “feelings check-ins” [19]. In the TF-ART protocol, each participant completed at least 13 collages or drawings and compiled in a hand-made book to describe his/her “life story” [19]. The use of art therapy in a traumatised child has also been described in a single case study [20].

Group art therapy has been described in the treatment of adolescent personality disorder, in an intervention where adolescents met weekly in two separate periods of 18 sessions over 6 months, with each session lasting 90 min, facilitated by a psychotherapist [21]. Sessions consisted of a short group conversation regarding events/issues during the previous week followed by a brief relaxing activity (e.g. listening to music), a period of art-making and an opportunity to explain their work, guided by the psychotherapist.

A long course of art psychotherapy over 3 years with a vulnerable female adolescent who presented with self-harm and later disclosed being a victim of a sexual assault has been described [22]. The young person described an “enemy” inside her which she had overcome in her testimony to her improvement, which was included in the published case study [22]. The approach of “art as therapy” has been described with children with bipolar disorder and other potential comorbidities, such as Asperger syndrome and attention deficit disorder, using the “naming the enemy” and “naming the friend” approaches [23].

The concept of the “transitional object”—a coping device for periods of separation in the mother–child dyad during infancy—has been considered in art therapy [24]. It was proposed that “transitional objects” could be used as bridging objects between a scary reality and the weak inner-self. Children brought their transitional objects to therapy sessions, and the therapy process aimed to detach the participant from his/her transitional object, giving him/her the strength to face life situations with his/her own capabilities [24].

Two studies of art therapy in children with adjustment disorders were included in our systematic review [25, 26]. Children attended two or three video-recorded sessions and were encouraged to use art materials to explore daily life events. The child and therapist then watched the video-recorded session and participated in a semi-structured interview that employed video-stimulated recall. The therapy aimed to transport the participant to a comfortable imaginary world, giving the child the possibility to create powerful, strong characters in his/her story, thus enhancing the ability to cope with life’s challenges [25, 26].

### Outcome measures and statistical analysis (Table 1)

Three articles on psychiatric disorders evaluated potential changes in outcome using an objective measure [18, 19, 22]. Two studies used the “The University of California at Los Angeles Children’s PTSD Index” (UCLA PTSD-I), which is a 20-item self-report tool [18, 19]. Statistical differences were evaluated by calculating the mean percentage change [18] and the ANOVA [19]. The 12-item “MacKenzie’s Group Climate Questionnaire” was used to measure the outcome of group art therapy in adolescents with personality disorder, and a significant reduction in conflict in the group was found [21]. However, the sample size was small, and there was no control group [21]. Many studies did not use highly recognised measures of outcome but relied instead on a comprehensive description of outcome or change after art therapy/psychotherapy, in case studies or case series [20, 22–27].

### Study design: children with psychiatric symptoms (Table 2)

We included seven studies in our review synthesis where art therapy or art psychotherapy was used as an intervention for psychiatric symptoms—many of these studies occurred in paediatric hospitals, where children were being treated for other conditions. Two of these studies were non-randomised controlled trials, one of which was waitlist controlled [28, 29], and the other five were quasi-experimental studies [30–34].

### Forms of intervention and assessment (Table 2)

Three articles described art therapy in paediatric hospital patients but varied in terms of therapy and underlying condition [28, 29, 33]. The effectiveness of art therapy on self-esteem and symptoms of depression in children with glaucoma has been investigated; a number of sensory-stimulating art materials were introduced during six individual 1-h sessions [33]. Short-term or single individual art therapy sessions have also been used in hospital aiming to improve quality of life [28, 29]. Art therapy has been provided to children with leukaemia; the children transformed unused socks into puppets called “healing sock creatures” [29]. Short-term art therapy prior to painful procedures, such as lumbar puncture or bone marrow aspiration, has also been described, using “visual imagination” and “medical play” with age-appropriate explanations about the procedure, with a cloth doll and medical instruments [28].

The remaining articles described the provision of art therapy to vulnerable patients, where the therapy aimed to increase self-confidence or address worries. Two studies focused on female self-esteem and self-concept, both using group activities [31, 32]. Hartz and Thick [32] compared two different art therapy protocols: art psychotherapy, which employed a brief psychoeducational presentation and encouraged abstraction, symbolization and verbalization and an art as therapy approach, which highlighted design potentials, technique and the creative problem-solving process, trying to evoke artistic experimentation and accomplishment rather than different strengths and aspects of personality [32]. Participants completed a known questionnaire about self-esteem as well as a study-specific questionnaire.

Coholic and Eys [34] described the use of a 12-week arts-based mindfulness group programme with vulnerable children referred by mental health or child welfare services, with a combination of group work and individual sessions [34]. Children were given tasks which included the “thought jar” (filling an empty glass jar with water and various-shaped and coloured beads representing thoughts and feelings), the “me as a tree” activity, during which the participant drew him/herself as a tree, enabling the participant to introduce him/herself, the “emotion listen and draw” activity which provided the opportunity to draw/paint feelings while listening to five different songs and the “bad day better” activity which involved painting what a “bad day” looked like, and then to decorate it to turn it into a “good day”. The research included quantitative analysis and qualitative assessment using self-report Piers-Harris Children’s Self-Concept Scale and the Resiliency Scales for Children and Adolescents [37, 38].

Kearns [30] described a single case study of art therapy with a child with a sensory integration difficulty, comparing teacher-reported behaviour patterns after art therapy sessions using kinaesthetic stimulation and visual stimulation with behaviour after 12 control sessions of non-art therapy; a greater improvement was reported with art therapy [30].

### Outcome measures and statistical analysis (Table 2)

Most of the studies on art therapy in children with psychiatric symptoms (but not confirmed disorders) used widely accepted outcome measures [29–34] (Table 2), such as self-report measurements including the 27-item symptom-orientated Children’s Depression Inventory or the Tennessee Self Concept Scale: Short Form [33, 35, 36]. The 60-item Piers-Harris Children’s Self-Concept Scale (2nd edition) and the Resiliency Scales for Children and Adolescents (RSCA) were used in a study on vulnerable children [34, 37, 38]. The Piers-Harris Children’s Self-Concept Scale is a widely used self-report measure of psychological health and self-concept in children and teens and consists of three global self-report scales presented in a 5-point Likert-type scale: sense of mastery (20 items), sense of relatedness (24 items) and emotional reactivity (20 items) [37]. A modified version of the Daley and Lecroy’s Go Grrrls Questionnaire was administered at group intake and follow-up, to rank various self-concept items including body image and self-esteem along a four-point ordinal scale in group therapy with young females [31, 39].

Some researchers created their own outcome measures [28–30, 33]. One study group created a mood questionnaire for young children—this was administered by a research assistant to patients before and after each therapy session, in their small wait-list controlled study [29]. Another group evaluated classroom performance using an observational system rated by the teacher for each 30-min block of time every day during the study [30]. The classroom study also used the “person picking an apple from a tree” (PPAT) drawing task—this was the only measurement tool in the studies we reviewed which assessed the features of the artworks themselves [30, 40]. Pre- and post-test drawings were evaluated for evidence of changes in various qualities over the course of the research period [30].

Hartz and Thick [32] used both the 45-items Self-Perception Profile for Adolescents (SPPA) [41] which is widely used and considered reliable, as well as the Hartz Art Therapy Self-Esteem Questionnaire (Hartz AT-SEQ) [32], which is a 20-question post-treatment questionnaire designed by the author, to understand how specific aspects of art therapy treatment affect self-esteem in a quasi-experimental study with group art therapy. Four of the seven articles performed statistical analysis of the data collected, using the Wilcoxon signed-rank test [31], Fisher’s *t* [32], MANOVA [34], and two-tailed Student’s *t* test [29].

### Assessment of bias

The QUADAS-2 assessment of bias for each study included in our systematic review synthesis can be seen in Table 3, with a summary of the results of the QUADAS-2 assessment for all included studies in our review in Table 4. Studies marked in green had a low risk of bias; those marked in red had a high risk of bias while those in yellow had an unclear risk of bias. Just two studies were found to have a low risk of bias [19, 29].

**Table 3:**
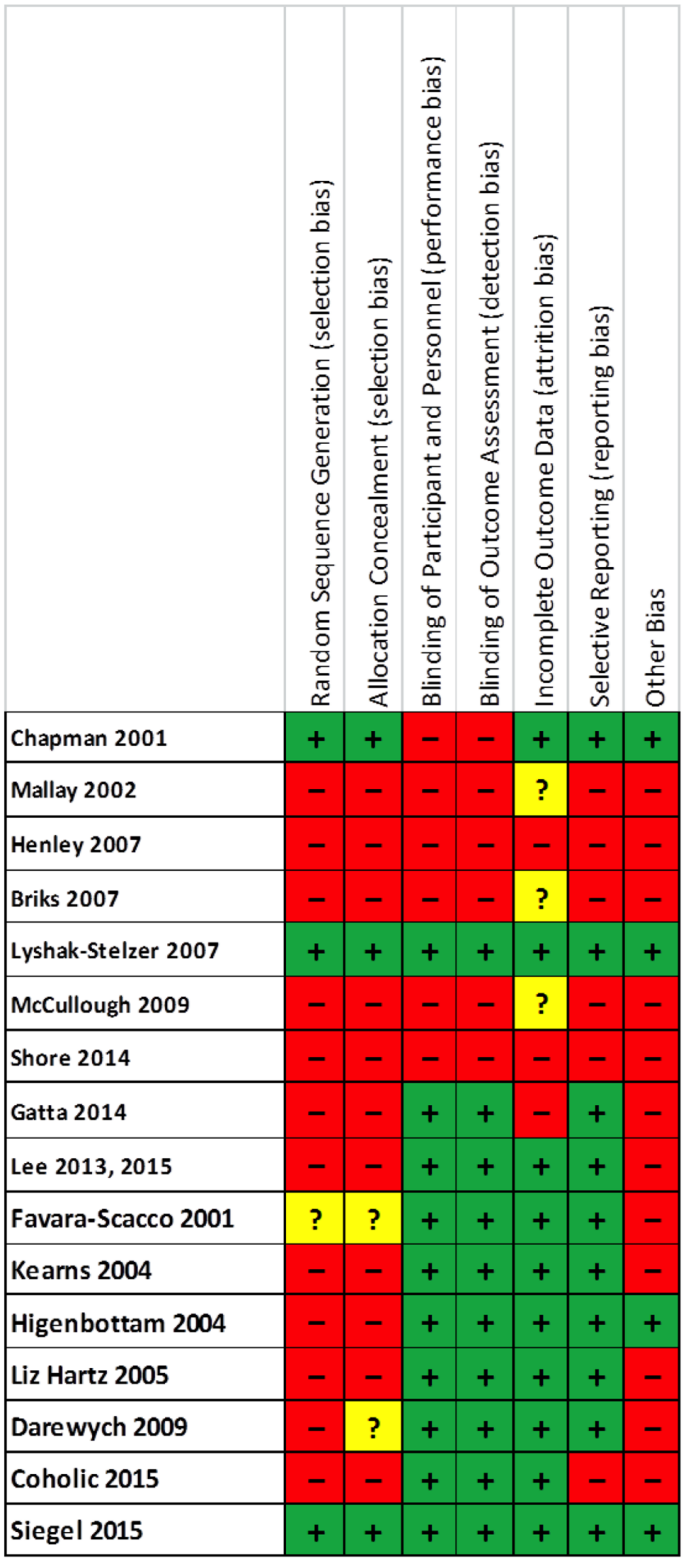
QUADAS-2 assessment of bias for each study included in the review

**Table 4:**
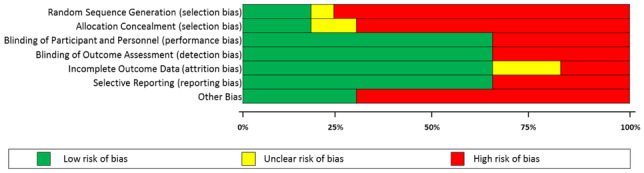
Summary of the Bias assessments (QUADAS-2) for all studies included in the review

## Discussion

We found extensive literature regarding the use of art therapy in children with mental health difficulties (*N* = 1273), with a large number of descriptive qualitative studies and cases studies, but a limited number of quantitative studies which we could include in our review synthesis (*N* = 17). The predominance of descriptive studies is not surprising considering that the field of art therapy and art psychotherapy has developed from the descriptive writings of Freud, Jung, Winnicott and others, and for many years, academic psychotherapy focused on detailed case descriptions rather than quantitative outcome studies. The numerous descriptive and qualitative publications generally described positive changes in participants undergoing art therapy, which may represent publication bias. Our aim was however to describe the quantitative evidence regarding the use of art therapy or art psychotherapy in children and adolescents with mental health difficulties, and we found a limited number of studies to include in our review synthesis. There were just two randomised controlled trials, no replication studies and insufficient information to allow for a meta-analysis. However, the articles in our review synthesis suggested that art therapy may have a positive outcome in various groups of patients, especially if the therapy lasts at least 8 weeks.

There is some evidence from controlled trials to support the use of art therapy in children who have experienced trauma [18, 19]. It should be noted that art therapy or art psychotherapy was delivered as individual sessions in most of the studies in our review, especially for children with a psychiatric diagnosis. A group approach to art therapy was used in some studies with vulnerable children such as children in need, female adolescents with self-esteem issues and female offenders [22, 31, 34]. However, the studies on group art therapy or psychotherapy are quasi-experimental studies of limited size, and it would be useful if larger, more robust studies such as randomised controlled trials could study the efficacy of group art therapy or group art psychotherapy.

Many of the studies included in our review synthesis ranked low in the Cochrane Risk of Bias criteria, with a high risk of bias. Our review synthesis highlights the heterogeneity of the studies—various methods of individual or group art therapy were delivered, with some studies delivering psychoanalytic-type interventions while others delivered interventions resembling cognitive behaviour therapy, delivered via art. The literature also showed a general lack of standardisation with regard to the duration of art therapy and outcome measures used. Despite this, the authors of many of the studies described common themes and hypothesised about the value of art therapy or art psychotherapy in improving self-esteem, communication and integration. The interventions often encouraged the child to re-enact or to process trauma, and the authors described improved integration, and therapeutic change or transformation of the young person. It appears that there were varied interventions in the studies in the review synthesis but that many studies had theoretical similarities.

### Strengths and limitations

We used clearly defined aims and followed PRISMA guidelines to perform this systematic review. However, we did not incorporate unpublished studies into our review and did not examine trial websites. By following strict exclusion criteria, we excluded studies on art psychotherapy and mental health where one or more participant commenced treatment before his/her eighteenth birthday and completed after the eighteenth birthday such as that by Lock et al. [42]. The Lock et al. [42] study may be of interest to those who are considering commissioning art therapy services for CAMHS, as it is a randomised controlled trial and suggests that art therapy may be a useful adjunct to Family-Based Treatment for adolescent anorexia nervosa in those with obsessive symptoms [42]. Our strict criteria also led us to exclude many studies where the primary focus was on educational issues including school behaviour or educational achievement—this is both a strength and limitation of our study. By excluding these studies, our systematic review can give useful information to CAMHS staff regarding the suitability of art therapy or art psychotherapy for children and adolescents with mental health difficulties. However, we note that a complete assessment of the effectiveness of art therapy or art psychotherapy in children would also include studies on the use of art therapy or art psychotherapy with children who have educational difficulties [43, 44], those with physical illness or disability, as well as describing the many studies on art therapy or art psychotherapy in children who are refugees or living in emergency accommodation. We focused our review on quantitative research, but there are many mixed-methods studies in art therapy and art psychotherapy, where qualitative studies analysis may be used to generate hypotheses, and quantitative methods are used to test the hypothesis. A complete analysis of the effectiveness of art therapy or art psychotherapy in children could include summaries of qualitative or mixed-methods studies as well as quantitative studies.

Meanwhile, it should be noted that there is considerable evidence for the effectiveness of psychotherapy in general [45, 46]. It has long been established that the common factors of alliance, empathy, expectations, cultural adaptation and therapist differences are important in the provision of effective psychotherapy [47]. Art therapy and art psychotherapy are more likely than the traditional talking therapies to provide these factors for those working with children.

## Conclusions and future perspectives

There is extensive literature which suggests that art therapy or art psychotherapy provide a non-invasive therapeutic space for young children to work through and process their fears, trauma and difficulties. Art has been used to enhance the therapeutic relationship and provide a non-verbal means of communication for those unable to verbally describe their feelings or past experiences. We noted that there is considerably more qualitative and case description research than quantitative research regarding art therapy and art psychotherapy in children. We found some quantitative evidence that art therapy may be of benefit in the treatment of children who were exposed to trauma. However, while there are positive outcomes in many studies regarding art therapy for children with mental health difficulties, further robust research and randomised controlled trials are needed in order to define new and stronger evidence-based guidelines and to establish the true efficacy of art psychotherapy in this population. It would be helpful if there were studies with standardised outcome measures to facilitate cross comparison of results.

## Data Availability

Data can be made available to reviewers if required.
